# Comparison of Effectiveness between Beclomethasone Dipropionate and Fluticasone Propionate in Treatment of Children with Moderate Asthma

**DOI:** 10.1097/WOX.0b013e3181f68d92

**Published:** 2010-10-15

**Authors:** Akefeh Ahmadiafshar, Mohsen Mogimi Hadji, Nima Rezaei

**Affiliations:** 1 Department of Pediatrics, Mousavi Hospital, Zanjan University of Medical Sciences, Zanjan, Iran; 2 Department of Pediatrics, Beheshti Hospital, Langrood, Iran; 3 Research Center for Immunodeficiencies, Children's Medical Center, Tehran University of Medical Sciences, Tehran, Iran; 4 Molecular Immunology Research Center; and Department of Immunology, School of Medicine, Tehran University of Medical Sciences, Tehran, Iran

**Keywords:** asthma, beclomethasone, fluticasone, inhaled corticosteroids, pulmonary function tests

## Abstract

Asthma is a common chronic disease. Beclomethasone dipropionate (BDP) and Fluticasone propionate (FP) are 2 inhaled corticosteroids that frequently be used in treatment of patients with asthma. In this study, the effectiveness of BDP and FP in management of asthmatic children was investigated. In this trial, 50 children with moderate persistent asthma were randomly selected to receive either BDP 600 *μ*g or FP 500 *μ*g for 3 months. Pulmonary function tests were measured in both groups at the beginning of study and monthly after treatment. Daily and night symptoms and consistency of drugs were also measured. There was significantly better FEV1 in patients receiving FP compared with the BDP group (*P *< 0.01). There was also statistically significant difference in patients receiving FP compared with BDP group in increment of FVC, FEV1/FVC, FEF25-75 (*P *< 0.005). Night symptoms were significantly improved in the FP group from the first month (*P *= 0.001), while improvement of daily symptoms in this group compared with the BDP group was found from the second month (*P *= 0.001). Although symptoms and pulmonary function tests results were improved in both groups receiving either FP or BDP, this study suggested that FP was more effective than BDP in controlling moderate asthma in children.

## 

Asthma is a chronic inflammatory disorder of the airways. There is a remarkable increase in prevalence of asthma worldwide, which makes it as one of the most common chronic diseases, especially in childhood [1,2]. The major characteristics of asthma include variable degrees of airflow obstruction, bronchial hyperresponsiveness and airway inflammation [3,4].

Inhaled corticosteroids (ICS) reduce both asthma symptoms and marker of asthma inflammation [5]. They are considered as the most potent and consistently effective long-term controller medications for asthma [6,7]. Fluticasone propionate (FP) and beclomethasone dipropionate (BDP) are 2 commonly ICS for the treatment of asthma.

There were several studies to compare the effects of these drugs in controlling asthma in different doses. This study was performed to compare the efficacy and acceptability of FP and BDP in nearly equal doses, in the treatment of asthmatic children with moderate persistent asthma.

## Patients and Methods

Fifty patients with diagnosis of moderate persistent asthma were enrolled in this study. The patients aged between 6 and 17 years and referred to the Allergy Clinic of Valie Asr Hospital (Zanjan, Iran) with symptoms of moderate persistent asthma within at least 6 months before enrollment. All these patients had symptoms of cough and wheezing (at night and after exercise) and a FEV1 of between 60-80% on the basis of NAEPP guideline [3]. Patients with other known causes of wheezing, such as cystic fibrosis and heart disorders, were excluded from this study. None of these patients had been receiving any ICS before the study.

It was an open label study in which the patients were randomly with unpredictable sequence, allocated into 2 groups. The first group received 250 *μ*g FP twice daily (2 puff BD) and the second group received 200 *μ*g BDP 3 times daily (4 puff TDS) for 3 months. All children were instructed to use ICS by mouthpiece spacer, provided by local company (Asan Nafas, Tehran Fanavar Teb, Iran). Pulmonary function tests (PFT) were done at the beginning of study and each month till the end of this study. Occurrence of daily and night symptoms and nonadherence of drug receiving were recorded to dairy card and assessed monthly.

Both ICS were registered trade mark of Cipla Company and prescribed open label; both of them had CFC propellant. Pulmonary function tests were done by one person who did not know any information about the kind of therapy. Spirometry was performed with a Jaeger Master Scope Spirometer (VIASYS Healthcare, Hochberg, Germany). The study protocol was approved by the Ethics Committee of Zanjan University of Medical Science; all patients or their parents gave written informed consent before enrollment. This study was also registered in the Iranian Registry of Control Trials (IRCT: 138812042976N2).

The results are presented as mean ± SD. The One-Sample Kolmogorov-Smirnov Test procedure was performed to check normal distribution of data by comparing the observed cumulative distribution function for a variable with a specified theoretical distribution. Comparisons of data between 2 groups were made using *t *test (for parametric data) and Mann-Whitney *U *test (for nonparametric data), *P *< 0.05 was considered as statistically significant. Repeated measurement test were also used for comparing individual responses to FP and BDP at 1, 2, and 3 months.

## Results

The patients aged 11.25 ± 4.3 years and 12.8 ± 4.4 years in FP and BDP groups, respectively. Baseline characteristics were similar in both groups for PFT results, night symptoms and asthma attacks at the beginning of the study (Table 1).

**Table 1:** Characteristics of Patients Who Received Fluticason Propionate (FP) and Beclomethason Dipropionate (BDP) at the Beginning of the Study

	FP	BDP	*P *Value
Number of studied patients	25	25	--
Male/female	12/13	15/10	0.39
Age; year (mean ± SD)	11.25 ± 4.3	12.8 ± 4.4	0.72
FEV1 (mean ± SD)	71.68 ± 5.76	69.48 ± 4.69	0.155
FVC (mean ± SD)	79.72 ± 7.61	72.60 ± 7.33	0.179
FEV1/FVC (mean ± SD)	0.74 ± 0.05	0.73 ± 0.04	0.852
FEF25-75 (mean ± SD)	49.6 ± 6.09	47.32 ± 6.34	0.201
Night symptoms*	6.32 ± 1.22	6.28 ± 1.24	0.880
Daily symptoms†	2.60 ± 1.16	2.80 ± 1.16	0.522

*Night problems per month. †Wheezing or cough per week need bronchodilator.

Although there was significant improvement of FEV1 in both groups in 3 consecutive months after treatment, such improvement in the FP group was significantly better than the BDP group in first (87.3 ± 11.1 vs. 79.8 ± 7.8, *P *= 0.008), second (91.5 ± 12.7 vs. 82.6 ± 8.8, *P *= 0.006), and third (90.3 ± 7.3 vs. 84.8 ± 7.1, *P *= 0.001) months. FVC and FEF25-75 in the FP group were also significantly improved compared with the BDP group in 3 consecutive months after treatment (Table 2). Although there was not significant difference on FEV1/FVC in first month after treatment between 2 groups, significant improvement of FEV1/FVC in second and third month of therapy with FP was found (*P *= 0.001). There were also significant improvement of night and daily symptoms in the patients used FP, compared with the patients used BDP (Table 2).

**Table 2:** Comparisons of Pulmonary Function Tests, Symptoms and Drug Nonadherence in the FP and the BDP Groups After First, Second, and Third Months of Treatment

Parameters	Flu Mean (SD)	BDP Mean (SD)	*P *Value
FEV1			
First	87.36(11.12)	79.84 (7.79)	0.008
Second	91.52 (12.67)	82.60 (8.76)	0.006
Third	90.32 (7.33)	84.84 (7.14)	0.001
FVC			
First	82.52 (8.13)	77.80 (8.08)	0.045
Second	89.36 (9.53)	79.56 (7.64)	0.001
Third	90 (10.64)	82.86 (5.88)	0.004
FEV1/FVC			
First	0.80 (0.046)%	0.79 (0.032)%	0.24
Second	0.85 (0.033)%	0.82 (0.017)%	0.001
Third	0.88 (0.029)%	0.83 (0.025)%	0.001
FEF25-75			
First	59.20 (6.38)	54.72 (5.79)	0.012
Second	66.96 (4.81)	62.36 (4.81)	0.003
Third	73.6 (2.53)	70.0 (4.25)	0.001
Night symptoms			
First	3.0 (1.08)	4.44 (0.92)	0.001
Second	2.12 (0.93)	3.28 (0.68)	0.001
Third	1.16 (0.85)	2.52 (0.51)	0.001
Daily symptoms			
First	1.16 (0.89)	1.68 (1.07)	0.065
Second	0.40 (0.50)	1.28 (0.94)	0.001
Third	0.20 (0.41)	0.48 (0.51)	0.039
Drug nonadherence			
First	0.72 (0.79)	1.48 (1.22)	0.023
Second	0.36 (0.63)	0.96 (0.84)	0.003
Third	0.4 (0.57)	0.64 (0.63)	0.156

The mean frequency of night symptoms per month before treatment was 6.3 in both groups. Although the frequency of such symptoms were significantly decreased after treatment with either FP or BDP group, there was significantly improvement in the FP group (*P *= 0.001). Mean frequency of daily symptoms per week in the FP group was also significantly improved compared with BDP group (Table 2).

There was significant difference in drug nonadherence in the first and second month, but there was no statistically different in third month (Table 2). We analyzed the changes between 2 groups during 3 consecutive months by repeated measurement test. We found significant difference in FEV1 (*P *= 0.001), FVC (*P *= 0.003), FEV1/FVC (*P *= 0.004), and FEF25-75 (*P *= 0.009). There was significant difference in night symptoms (*P *= 0.0001), daily symptoms (*P *= 0.009), and drug nonadherence (*P *= 0.001). We did not find any side effect or complication in both groups.

## Discussion

Inhaled corticosteroids are effective drugs for treatment of asthma [6,7]. This study showed that both FP and BDP can improve the symptoms and PFT results. However, the degree of improvement in daily and night symptoms and PFT results among patients treated with FP was significantly better than the BDP group. This could be because of differences in pharmacokinetics and pharmacodynamics of FP and BDP [8].

Although the results of our study was in agreement with some previous studies toward benefit of FP than BDP,[9-11] the studies by Nong et al [12] and Karakoc et al [13] did not find any significant difference in lung function improvement and symptom scores in asthmatic children receiving either BDP or FP. However, these studied might be limited by the fact that they considered half dose of FP than BDP in their study [13]. Other studies showed CFC free product of BDP compared with FDP (or beclomethasone/formeterole vs. fluticasone/salmeterole) administration were equally effective for improving asthma control in children with mild to moderate asthma at the same daily dose [14,15]. Molimard et al showed that efficacy of beclomethasone extrafine aerosol was significantly better than fluticasone and budesonide [16]. In our study, we used CFC-propelled BDP and did not find better response by this product.

BDP was the first ICS, substituted for systemic corticosteroids in the treatment of chronic asthma [8]. It is the cheapest ICS currently available at both low and high doses and may remain so even, when CFC-propelled products are excluded,[17] which might lead to good acceptance of BDP in our patients. It should be noted that similar results were achieved in drug administration in BDP and FP groups in third month, despite greater daily doses of BDP than FP.

ICS are effective potent medication in asthma with fewer side effects,[7,18-20] but different studies showed increasing evidence of adverse effects in patients treated with Fluticasone than Beclomethasone [10,21-24]. However, our patients did not have any problem such as hoarseness, pharyngitis, or candidiasis of oropharyngeal area during the study period. This may be because of instruction of patients in using spacer and mouth rinsing after drug prescribing and the short course of therapy.

It should be emphasized that conclusion from this study is limited by the fact that we performed an open label study, and we did not include a placebo control as it was unethical to keep a group untreated. Indeed we compared CFC-BDP (with its lower efficacy compared with FP at equivalent doses given TDS (raising issues of adherence) with FP given at similar doses but BD. However, BDP is a short acting ICS in comparison to FP, and routinely prescribed more than 2 times a day, but FP could be even prescribed once per day [18]; and thus, BDP was prescribed more times than FP in our study. By the way, the patients recruited and the methodology used should be taken into consideration before generlizability of the findings.

In conclusion, this study showed that FP in a daily dose of 500 *μ*g results in a significantly greater increase in PFT results and improvement of symptoms than BDP at the dose of 600 *μ*g in children with moderate asthma. Further studies with different doses of these drugs and different types of ICS are recommended to choose the best effective ICS with fewer side effect and better acceptance for patients.
